# Correlation between compliance and results of brace treatment in juvenile and adolescent idiopathic scoliosis

**DOI:** 10.1186/1748-7161-9-S1-O28

**Published:** 2014-12-04

**Authors:** Angelo Gabriele Aulisa, Vincenzo Guzzanti, Marco Giordano, Marco Fuiano, Paolo Pizzetti, Lorenzo Aulisa

**Affiliations:** 1 UOC Orthopaedic and Traumatology Childrens Hospital Bambino Gesu, Rome, Italy; 2 Department of Orthopaedics Universita Cattolica del Sacro Cuore, Rome, Italy; 3 Independent practitioner, Rome, Italy

## Background

In the last years the literature suggest that the efficacy of bracing to reduce the curve progression and the surgery is good in compliant patients. Some of the studies applied a sensor other standardised form. Therefore the lack evidence of the effectiveness of bracing may partly be explained by poor compliance.

## Aim

The aim of the present study was to prospectively evaluate the association between compliance of brace wear and progression of the scoliotic curve including the surgical rate in patients with idiopathic adolescent and juvenile scoliosis treated with PASB, Lyon brace and Milwaukee.

## Design

Prospective study from 1424 patients treated for idiopathic scoliosis between 1995 and 2013.

## Methods

Fulfill the inclusion criteria (age between 4-12 years, full-time prescription) 683 patients. Of these, 505 patients had a definite outcome, 107 have abandoned treatment and 71 are still in treatment . The minimum duration of follow-up was 24 months. Three outcomes were distinguished in agreement with SRS criteria: curve correction, curve stabilization and curve progression. Wearing of the brace was assessed by one orthopaedic surgeon (LA) and scored on a standardized form. Scoring was based upon questioning the patient and the parents. The Compliance was reported as: Complete (used as prescribed); Incomplete A (removed for 1 month); Incomplete B (removed for 2 months); Incomplete C (removed for school time, about 6-8 hours); Incomplete D (only the night). The Kruskal Wallis and Spearman Rank Correlation tests have been used as statistical tests.

## Results

The results from our study showed that at follow-up the compliance was: Complete 58%; Incomplete A 6.3%; Incomplete B 11.7%; Incomplete C 16.2%; Incomplete D 7.7%.

The 505 patients with a definite outcome Cobb mean value was 29.8 ± 7.5 SD at beginning and 17.1 ±10.9 SD at follow-up. Perdriolle was 13.2 ± 5.6 SD at beginning and 7.6 ± 4.8 at follow-up.

Curve correction/stabilization was accomplished in 291/293 of Complete, 32/32 Incomplete A, 55/59 Incomplete B, 79/82 Incomplete C, 37/39 Incomplete D. Surgery Referral was 3/293 of Complete, 0/32 Incomplete A, 0/59 Incomplete B, 2/82 Incomplete C, 2/39 Incomplete D.

## Conclusion

We conclude that the risk for curve progression and surgery are reduced in patients with high brace compliance. In particular the abandon of treatment for only 1 month is not influence for the good results while wearing the brace only the night time increased curve progression.

